# Correction: Martin et al. The FeetMe^®^ Insoles System: Repeatability, Standard Error of Measure, and Responsiveness. *Sensors* 2024, *24*, 6043

**DOI:** 10.3390/s25103061

**Published:** 2025-05-13

**Authors:** Nathan Martin, Fabien Leboeuf, Didier Pradon

**Affiliations:** 1 Pôle Parasport—ISPC Synergies, CHU Raymond Poincaré, APHP, 92380 Garches, France; nathanmartin.coe@outlook.fr; 2 Service de Médecine Physique et Réadapatation Locomotrice et Respiratoire, CHU Nantes, Nantes Université, 44093 Nantes, France; fabien.leboeuf@chu-nantes.fr; 3 Movement-Interactions-Performance (MIP), EA 4334, CHU Nantes, Nantes Université, 44000 Nantes, France; 4 U1179 Endicap, UVSQ, 78000 Versailles, France

## Text Correction

In the original publication [1], there was a mistake in Section 2.1. The sentence “This evaluation was carried out within the framework of a database of healthy subjects for the “No-Barriers” study, aimed at identifying the barriers to physical activity for people with disabilities (NCT05294068).” should read as “This evaluation was carried out within the framework of a database of healthy subjects for a research protocol on the effect of repeated botulinum toxin injections [Committee for the protection of persons île-de-France 2015-A01671-48, NCT02699775].”.

The correct “Institutional Review Board Statement” appears as follows:

**Institutional Review Board Statement:** The study was conducted in accordance with the Declaration of Helsinki and approved by the Committee for the protection of persons île-de-France (2015-A01671-48).

The authors state that the scientific conclusions are unaffected. This correction was approved by the Academic Editor. The original publication has also been updated.
